# MicroRNA profiles of prostate cancer bone metastases and miR-375 as an inhibitor of the aggressive metastasis subtype MetB

**DOI:** 10.1038/s41598-026-67032-9

**Published:** 2026-08-25

**Authors:** Helena Järemo, Julius Semenas, Sofia Halin Bergström, Elin Thysell, Marie Lundholm, Eva Freyhult, Andreas Josefsson, Karin Welén, Camilla Thellenberg Karlsson, Sead Crnalic, Anders Bergh, Maria Brattsand, Pernilla Wikström

**Affiliations:** 1 Department of Medical Biosciences, Pathology, Umeå University, 901 87 Umeå, Sweden; 2 Science for Life Laboratory, Department of Cell and Molecular Biology, National Bioinformatics Infrastructure Sweden, Uppsala University, Uppsala, Sweden; 3 Department of Diagnostics and Intervention, Urology and Andrology, Umeå University, 901 87 Umeå, Sweden; 4 Wallenberg Centre for Molecular Medicine, Umeå University, 901 87 Umeå, Sweden; 5 Department of Urology, Sahlgrenska Center for Cancer Research, Institute of Clinical Sciences, Sahlgrenska Academy, University of Gothenburg, Gothenburg, Sweden; 6 Department of Diagnostics and Intervention, Oncology, Umeå University, 901 87 Umeå, Sweden; 7 Department of Diagnostics and Intervention, Orthopaedics, Umeå University, 901 87 Umeå, Sweden

**Keywords:** Prostate cancer, Metastasis, Subtypes MetA-C, microRNA, miR-375, Cancer, Oncology

## Abstract

Novel treatments for metastatic prostate cancer (PC) are needed. Molecular subtypes of bone metastatic PC (MetA-C) were recently identified, with the proliferation-driven MetB subtype linked to very poor prognosis. Non-coding microRNAs (miRs) regulate gene expression and constitute potential therapeutic targets. This study aimed to explore miR profiles in bone metastases in relation to the MetA-C subtypes, with focus on the aggressive MetB subtype. Genome-wide microarray analysis of clinical bone metastasis samples (n = 96, 62 patients) identified miR-375 to be inversely associated with the MetB subtype. The metastatic C4-2B cell line was transduced to overexpress or sequester miR-375 and effects were evaluated by microarray analysis, cell proliferation/adhesion assays, and tumor growth in mice. miR-375 overexpression in C4-2B altered transcription profiles and shifted the phenotype from being MetB-like to becoming MetA-like. In cell culture, miR-375 overexpression reduced cell proliferation, increased adhesion, and downregulated *YAP1* expression among other direct gene targets, while miR-375 inhibition had opposite effects. In mice, miR-375 overexpression reduced tumor establishment and growth. Our data link clinical and experimental findings of miR-375 being a potential inhibitor of the aggressive MetB subtype in PC.

**Supplementary Information:**

The online version contains supplementary material available at 10.1038/s41598-026-67032-9.

## Introduction

Prostate cancer (PC) is a major health issue world-wide^1^. Localized PC can be cured by surgery or irradiation, but metastatic PC remains lethal. Current treatments for metastatic PC rely on androgen deprivation therapy (ADT) and androgen receptor (AR) inhibition, often in combination with cytotoxic drugs, but outcomes vary, and new therapeutic strategies and prognostic and treatment-predictive biomarkers are needed.

We previously defined three transcriptomic subtypes of PC bone metastases (MetA-C)^2,3^. MetA, the most common subtype, is characterized by high AR activity, as defined by the expression levels of *AR* and AR-regulated genes such as *KLK2, KLK3, TMPRSS2, SLC45A3* and others, low proliferative activity, and a favourable response to ADT. MetB is characterized by low AR activity despite generally retained AR expression, high proliferative activity, and poor response to ADT and AR-targeted therapies, whereas MetC is characterized by a stromal-related phenotype^2,3^. Notably, however, most metastases are heterogeneous for the MetA-C subtypes^3^.

A study of transcriptomic data from 769 metastatic PC patients identified the MetB profile among the few gene expression pathways that independently predicted adverse outcome^4^, and MetA-C classification of primary biopsies could potentially guide treatment decisions already at metastasis diagnosis^5^. The proliferation-driven MetB subtype has been associated with *RB1* aberrations and specific DNA methylation profiles^3,6^, but the pathophysiology of the MetA-C subtypes remains largely unexplored.

MicroRNAs (miRs) are short non-coding RNAs that repress translation and fine-tune expression of multiple mRNAs^7^. Through this broad regulatory capacity, they hold promise both in diagnostics and therapeutics^8,9^. Differential miR profiles have been observed in primary PC, and their potential as biomarkers extensively studied^10,11^. However, the miR landscape of PC bone metastases is scarcely examined^12^, and the role in MetA-C subtype development remains completely unknown.

The study aimed to find functional links between miRs and the metastasis subtypes MetA-C, with particular focus on the aggressive MetB subtype. Based on the inverse association observed between miR-375 and the aggressive MetB subtype, and on the potential role of miR-375 as a regulator of epithelial cell plasticity^13,14^, miR-375 was taken forward for experimental exploration as a MetB inhibitor.

## Materials and methods

### Patient samples

Bone metastasis tissue samples (n = 96) were collected from 62 PC patients at Umeå University Hospital or Sahlgrenska University Hospital between 2003 and 2019 (Table 1). The cohort has been previously described in detail^3^. Fresh-frozen tissue was collected from patients at metastatic surgery due to spinal cord compression (n = 49) or as core biopsies from iliac crest (n = 13). Replicate samples were obtained from 15 patients and from five patients longitudinal sampling was done. The study was approved by local ethic review boards of Umeå University (Dnr 03-185 with amendments Dnr 04-026 M, Dnr 2013-57-31 M) and Gothenburg (Dnr 455-11 with amendment 2019-05732). The study was conducted in accordance with the Declaration of Helsinki and in accordance with relevant guidelines and regulations. All patients gave their informed consent.

**Table 1:** Clinical characteristics and MetA-C subtypes of patients and metastasis samples.

	Median (range)
Age at diagnosis (yrs)	70 (52–85)
Age at metastasis sampling (yrs)	74 (61–87)
Serum PSA diagnosis (ng/ml)	120 (2.1–10,000)
Serum PSA metastasis sampling (ng/ml)	160 (1.4–12,000)
Follow-up after diagnosis (yrs)	5.4 (0.23–19)
Follow-up after ADT (yrs)	3.9 (0.22–17)
Follow-up after metastasis sampling (yrs)	1.8 (0.025–13)

	Patients N (%)	Samples N (%)
Gleason score at diagnosis:		
6	5 (8%)	
7	12 (19%)	
8–10	24 (39%)	
Not available	21 (34%)	
Primary treatment:		
Radical prostatectomy	9 (14%)	
Radiotherapy	8 (13%)	
Castration	45 (73%)	
Castration therapy^a^:		
None (hormone-naïve)	15 (24%)	
Short-term^**b**^	10 (16%)	
Long-term (CRPC)	37 (60%)	
Treatment for CRPC:		
Bicalutamide	22 (35%)	
Chemotherapy	11 (18%)	
Abiraterone acetate	6 (10%)	
Enzalutamide	5 (8%)	
Ra223	3 (5%)	
Zoledronic acid/Denosumab	4 (6%)	
Metastasis site:		
Spine (surgery)	49 (79%)	77 (80%)^d^
Iliac crest (core biopsies)	13 (21%)	19 (20%)^d^

	Patients N, Median (range)	Samples N, Median (range)
Metastasis subtype^c^:		
MetA	48, 0.84 (0.41–1.0)	72, 0.82 (0.41–1.0)
MetB	7, 0.75 (0.34–1.0)	12, 0.76 (0.34–1.0)
MetC	7, 0.54 (0.44–0.68)	12, 0.58 (0.44–0.79)

^a^Castration therapies given prior to collection of metastasis tissue samples included surgical ablation, LHRH/GnRH agonist therapy and bicalutamide treatment. ^**b**^Castration therapy for 1 day to 3 months before metastasis tissue sampling. ^c^Metastasis subtype estimated as described in the method section and presented as numbers per dominating subtype (N) with the median MetA-C similarity and range given. ^d^Replicate samples were obtained from 15 metastases (2–6 replicates/patient) and from five patients longitudinal sampling was done at 1-3 occasions.

### Cell lines

The C4-2B, 22Rv1, PC-3, and DU145 cell lines (RRIDs: CVCL-4784, -1045, -0035, -0105) were acquired (Cat#CRL-3315, CRL-2505, CRL-1435, HTB-81) from the American Type Culture Collection (ATCC, Manassas, VA, US) and cultured according to protocols. The cell lines were authenticated by short tandem repeat analysis (ATCC) and thereafter kept at low passages. Cells were confirmed mycoplasma-free at different time-points, either by PCR evaluation (IDEXX, Kornwestheim, Germany) or by in house MycoStrip testing (InvivoGen, San Diego, CA, USA).

To overexpress miR-375, C4-2B cells were transduced by the shMIMIC Inducible Lentiviral Tet-on 3G vector with tetracycline-inducible expression of hsa-miR-375 microRNA mEF1α Turbo GFP (Cat#VSH6904-224649615, Horizon Discovery, Cambridge, UK), according to manufacturer’s recommendation. To express an inducible non-target control sequence (NTC), cells were transduced with SMARTvector Inducible NTC mEF1α TurboGFP (Cat#VSC6574, Horizon Discovery). Stable clones were selected with puromycin (Thermo Fisher Scientific, Waltham, MA, USA). The established C4-2B^375^ and C4-2B^NTC^ clones were maintained in tetracycline-depleted FBS (Gibco, Thermo Fisher Scientific). For miR-375 inhibition, cells were transduced with a constitutively expressed pEZX-AM03 anti-miR-375 construct (Cat#LPP-HmiR-AN0466-AM03-100S) or a scramble control sequence control (Cat#LP503-100, Genecopoeia, Rockville, MD, USA) and selected with hygromycin (Thermo Fisher Scientific). Dihydrotestosterone (DHT) (Merck, Darmstadt, Germany) and doxycycline (Dox) (Stemcell Technologies, Cambridge, UK) were added as indicated.

### miR and mRNA profiling

Short RNAs were isolated from clinical samples using the Allprep DNA/RNA/Protein Mini Kit followed by the RNeasy MinElute Cleanup kit (Qiagen, Hilden, Germany) and profiled on GeneChip™ miRNA 4.0 covering miRBase release 20 (n = 2578) (Thermo Fisher Scientific). RNA was poly(A)-tailed, biotin-labeled using the FlashTag™ Biotin HSR Kit (Thermo Fisher Scientific), hybridized, washed, stained, and scanned on a GeneTitan™ Multi-Channel Instrument, according to protocol (Thermo Fisher Scientific). Cell line RNA was extracted using the Allprep DNA/RNA/miRNA Universal Kit (Qiagen) and profiled as described above for miRs and by using the GeneChip™ WT PLUS Kit in combination with Clariom D arrays for mRNA, according to protocols (Thermo Fisher Scientific). Previously generated mRNA profiles of bone metastases (GSE189343) were available^3^.

### Data processing and analysis

For miR analysis, raw probe values were normalized using the Robust Multi-array Average method in the Transcriptome Analysis Console Software (v4.0, Thermo Fisher Scientific). Further processing was performed in R (v4.2.2) in RStudio (v3.16). In the clinical samples, probes with detection *P* ≤ 0.05 in ≥ 5 samples were retained, batch-corrected (array order, site of metastases, hospital), and filtered for variability (SD ≥ 0.81).

Differential expressions were analyzed with limma (v3.64.1) (https://bioconductor.org/packages/release/bioc/html/limma.html). Hierarchical clustering was performed with hclust (v4.2.2) and heatmaps with ComplexHeatmap (v2.14.0) (Bioconductor.org). Functional enrichment analysis was performed at the MetaCore platform (adj.*P* ≤ 0.05) (Clarivate, London, UK). Predicted miR-375 targets were obtained from TargetScanHuman release 8.0 (https://www.targetscan.org/vert_80/).

### Classification of the MetA-C subtypes and estimation of tumor cell content

The MetA-C subtypes of metastasis samples and cell lines were estimated using CIBERSORTx (https://cibersortx.stanford.edu/)^15^ based on expression levels of 157 predefined MetA-C-differentiating gene transcripts as previously described^3^, and reported by similarity to MetA-C (range 0–1) and by the dominant subtype. The MetA-C subtypes of the clinical samples are given in Table 1 and Table S1. The patients were classified into subtypes based on mean MetA-C values if replicate metastasis samples were analyzed (Table 1, Table S2). Tumor cell content was estimated with ESTIMATE (v1.0.13) ( https://bioinformatics.mdanderson.org/public-software/estimate/) ^16^.

### In vitro experiments

In 2-dimensional (2D) cell growth experiments, 4000 cells/well were seeded in 96-well plates for 4 days. In 3D assays, 500 cells/µl were embedded in 3 µl-drops of Matrigel (Cat#356231, Merck) and cultured for up to 7 days + /- Dox. Media was changed every other day. Growth was measured using 2D/3D CellTiter-Glo™ Cell Viability Assays (Promega, Madison, WI, USA) and luminescence was read on Spectramax i3x (Molecular Devices, San Jose, CA, USA). Cell adhesion was evaluated with Vybrant Cell adhesion assay (Thermo Fisher Scientific), according to the manufacturer’s description. Cells were labeled with calcein and plated (5 × 10^5^ cells/well) in 96-well plates coated with a mix of fibronectin, collagen and albumin (Gentaur, Eersel, The Netherlands).

### Animal studies

Inducible C4-2B^375^ cells (6 × 10^6^ cells in cell media/Matrigel, 1:1, 100 µl) were injected subcutaneously into the flank of Balb/c Nude mice (Charles River Laboratories, MA, USA) (n = 31). Tumor volumes were measured with a caliper twice weekly (V = l × (w^2^)/2). At ~ 100 mm^3^, mice were randomized to control water containing 2% sucrose (n = 6) or Dox-water (0.2 mg/ml dox, 2% sucrose) (n = 6). In parallel, wild-type (WT) C4-2B cells were injected (n = 7) and mice were given drinking-water as described above (n = 3 + 3). Animals without palpable tumors within 10 weeks were excluded. Mice were euthanized at predefined endpoints (tumor volume 1000 mm^3^ and/or a maximum body weight loss of 20%) or after 24 days. At the time of euthanasia, mice were anesthetized via intraperitoneal injections of Medetomidine (0.75 mg/kg), Midazolam (5 mg/kg), and Fentanyl (0.05 mg/kg), followed by cardiac removal. Tumors were processed as fresh-frozen and formalin-fixed paraffin-embedded (FFPE). Animal experiments were approved by the Umeå Ethical Committee for Animal Studies (A16-2020) and performed in accordance with relevant guidelines and regulations.

### RT-qPCR

Transcript levels of YAP1 were quantified by reverse-transcription (Superscript VILO Master Mix), and real-time qPCR (QuantStudio™ 6 Flex) with GAPDH as endogenous control, using TaqMan® Gene Expression Assays (Cat#Hs00371735_m1, Cat#Hs99999905_m1) according to protocols (Thermo Fisher Scientific). miR-375 was analyzed with TaqMan® MicroRNA reverse transcription kit (Cat#4366596) and the Taqman™ MicroRNA Assay hsa-miR-375 (Assay ID 000564) with RNU48 and U47 as endogenous controls (Assay IDs 001006, 001223. Relative levels were calculated by the comparative ΔΔCT-method.

### In situ hybridization

Cells in 3D cultures (500 cells/µl in 40-µl Matrigel drops) were grown + /- Dox for 7 days, fixed in formalin, embedded in Histogel (Epredia, Kalamazoo, MI, USA) and processed as FFPE. In situ hybridization for miR-375 was performed on sections of FFPE bone metastasis tissue and 3D cell cultures using the Discovery Ultra platform (Roche Diagnostics, Basel, Switzerland), the miR-375 miRCURY detection probe (Cat#33911) and the miRCURY LNA miRNA ISH Buffer Set (Cat#339450), with scramble and U6 as negative and positive controls, according to protocol (Qiagen).

### Immunohistochemistry

Sections of FFPE tumor tissue obtained from the animal experiments and cells in 3D culture (processed as described above) were immunostained on the Discovery Ultra platform for Ki67 (anti-Ki-67 (30–9) Rabbit Monoclonal antibody, ready to use, Cat#790-4286, Roche Diagnostics) and YAP1 (Anti-YAP rabbit monoclonal antibody, diluted 1:200, Cat#ab52771, Abcam, Cambridge, UK). Antigen retrieval was performed with Ultra CC1 (Ki67) and CC2 (YAP1) buffer and detection with OmniMap anti-Rabbit HRP and ChromoMap DAB, according to protocol (Roche Diagnostics). The percentage (%) of Ki67 positive tumor cells (~ 7000 cells/sample) was quantified in QuPath^17^. The YAP1 immunoreactivity was quantified based on the area (0: 0%, 1: 1–25%, 2: 26–50%, 3: 51–75%, and 4: 76–100%) and intensity (0: negative, 1: week, 2: moderate, and 3: intense staining) of positive cells. An immunoreactivity score (0–12) was obtained by multiplying values for distribution and intensity.

### Western blotting

Proteins from cells in culture + /- Dox were extracted in RIPA buffer (Thermofisher Scientific), separated (20 µg/sample) by SDS-PAGE, and transferred to nitrocellulose membranes, according to standard procedures. Membranes were incubated with antibodies targeting YAP1 (1:10 000) or β-Actin (1:5000, Cat#A5441, Merck) followed by secondary goat anti-rabbit HRP (1:10 000, Cat#P044801-2, Agilent, Santa Clara, CA, USA) or anti-mouse HRP (1:10 000, Cat#P0447, Agilent) antibodies, respectively. Signals were detected with ECL Reagent (Fisher Scientific, Leicestershire, UK) and quantified by the ChemiDoc Touch Imaging System (BIO-RAD, Hercules, CA, USA).

### Statistical analysis

Associations (*adj.P* ≤ 0.05) between miRNA expression and clinical or sample-related variables were evaluated using linear regression analysis in R (v4.2.2, Rstudio v3.16), with limma (3.54.2) and nmle (3.1–160), adjusting for batch effects of array running order, anatomical site, hospital, conditionally for estimated tumor cell content, and reported as regression coefficients (β-values). The replicate correlation was estimated using the ‘duplicateCorrelation’ function. Cancer-specific survival was assessed by log-rank test, based on replicate means per patient, with PC death as event and other causes as censored events. When longitudinal metastasis sampling was performed for a patient who received additional therapy in between samplings, the patient was included twice in analyses of clinical characteristics. Groups were compared by the unpaired t-test for continuous variables and by the Chi-square test for categorical variables. Tumor growth in mice over time was compared by the Mixed effect model (GraphPad Prism v.10.4.2). *P* ≤ 0.05 was considered significant.

## Results

### miR expression in relation to the MetA-C subtypes

Microarray analysis of 96 bone metastasis samples from 62 PC patients assessed 2578 human miRs (miRBase 20), of which 964 were significantly detected in ≥ 5 samples. After batch correction, 459 miRs with high expression variation were selected for further analysis (Table S1). Unsupervised hierarchical cluster analysis based on these 459 miRs identified four main patient clusters that showed no clear association with the clinical variables in Table 1 (Fig. S1, Table S2).

**Fig. 1:**
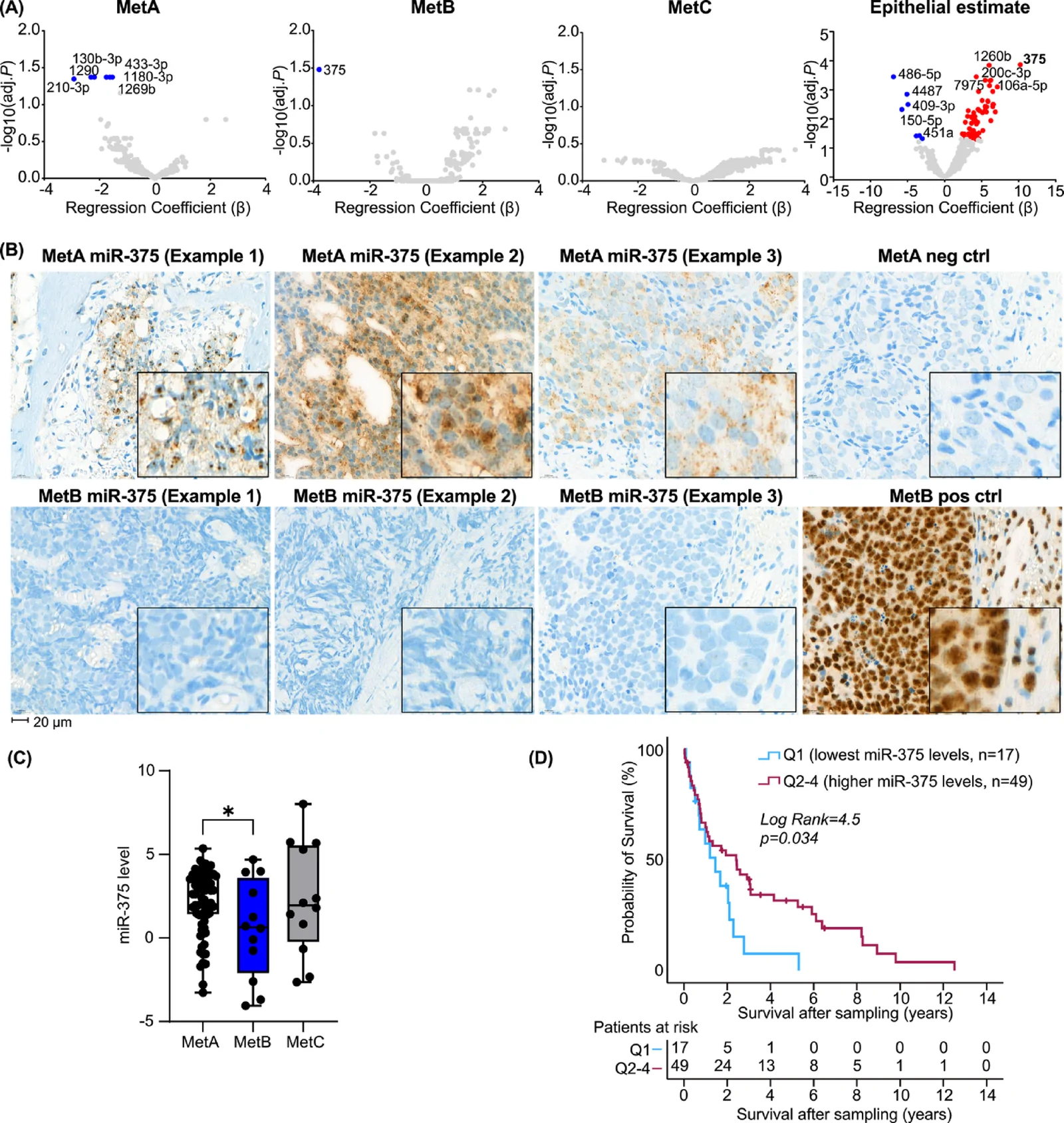
Low expression of miR-375 in bone metastases of the MetB subtype. (**A**) Volcano plots of miRs showing positive (red) or negative (blue) associations with the metastasis subtypes MetA, MetB or MetC, as well as the estimated epithelial tumor cell content (*adj.P* ≤ 0.05). Significantly associated or top 5 miRs are abelled. (**B**) In situ hybridization analysis of miR-375 in 6 metastases showing positive cytoplastic staining of variable intensity in MetA tumor cells and negative staining in MetB. Negative (scramble-probe) and positive (U6 small nuclear RNA) control staining, respectively, were performed in tissue sections corresponding to positive (MetA) and negative (MetB) miR-375 staining as indicated. (**C**) Relative miR-375 levels in metastasis samples of dominant subtype MetA (n = 72), MetB (n = 12), and MetC (n = 12). *P < 0.05. (**D**) Kaplan–Meier survival analysis of miR-375 levels, dichotomized into quartile 1 (Q1: the lowest 25^th^ percentile) and Q2-4, in relation to patient survival after metastasis sampling.

To identify miRs associated with the aggressive, proliferation-driven MetB subtype^2,3^, and its particularly poor prognosis (Fig. S2), we performed association analysis of the 459 miRs and the sample MetA-C subtypes (Table S3). An inverse association was observed between MetB and miR-375 (*β* = − 3.8, adj.*P* = 0.033) (Fig. 1A). MetA showed inverse associations with miR-130b-3p, -1180-3p, -1290, -1269b, -433-3p, and -210-3p (adj.*P* < 0.05), while no miR was significantly associated with MetC (Fig. 1A). MiR-375 was strongly associated with estimated tumor cell content (*β* = 10, adj.*P* = 0.00014) (Fig. 1A). In situ hybridization analysis was performed to explore miR-375 staining in 6 metastases selected to represent MetA (n = 3) and MetB (n = 3) cases with generally high and low levels, respectively. Cytoplasmic staining of variable intensities was confirmed in MetA tumor cells, while MetB tumor cells were negative (Fig. 1B). Accordingly, the median miR-375 level was significantly lower in metastases classified as MetB compared to cases classified as MetA, although high variance was observed in all subtypes (Fig. 1C). Patients with the lowest miR-375 metastasis levels (Q1) had the worst outcome (Fig. 1D). Based on this, and on the documented role of miR-375 as a potential regulator of epithelial cell plasticity^13,14^, miR-375 was selected for experimental exploration as an inhibitor of the MetB phenotype.

### The C4-2B as a model to study MetA–MetB plasticity and miR-375 function

**Fig. 2:**
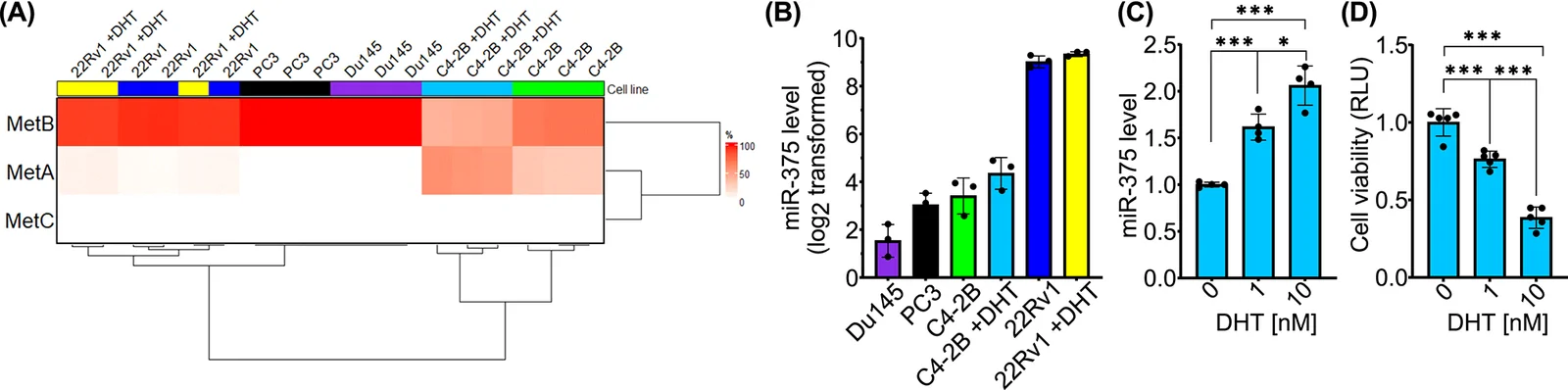
The C4-2B cell line as a model system for MetA and MetB subtype plasticity. (**A**) Similarity of the prostate cancer cell lines (22Rv1, PC-3, DU145, C4-2B) to MetA-C subtypes according to classification based on 157 differentiating transcripts (Clariom D array analysis, 48 h +/− 1 nM dihydrotestosterone (DHT), red and white; 100% and 0% similarity, respectively). (**B**) Relative miR-375 levels of the samples in A (GeneChip™ miRNA 4.0 array analysis). (**C**–**D**) Relative miR-375 levels (**C**) and cell viability (**D**) in C4-2B cells after treatment with 0, 1 or 10 nM DHT for 4 days (qRT-PCR and CellTiterGlo). Data is shown from one representative experiment with replicates as indicated; experiments were independently repeated 2 (**C**) and 3 (**D**) times with similar results. RLU, relative luminescence units. Bars indicate mean ± SD, individual values shown. *P < 0.05, ***P < 0.001 (unpaired t-test).

Global miR and mRNA profiles of the AR-positive (C4-2B, 22Rv1) and AR-negative (DU145, PC-3) cell lines were examined by microarray analysis, and their MetA-C subtypes were determined based on expression levels of a predefined set of MetA-C differentiating transcripts (Table S4). To capture MetB to MetA plasticity related to AR activation, AR positive cell lines were stimulated with DHT. At baseline, C4-2B showed about 70% and 30% similarity to MetB and MetA profiles, respectively, while 22Rv1, PC-3, and DU145 closely resembled MetB (94–100%) (Fig. 2A) (Table S4). No cell line showed any similarity to MetC (0%) (Fig. 2A) (Table S4). By DHT stimulation, C4-2B shifted from dominating MetB (70%) to dominating MetA (56%), whereas 22Rv1 was unaffected (Fig. 2A) (Table S4), plausibly due to its high expression levels of constitutively active AR variants^18^, driving AR activity and cell proliferation independently of androgen levels. Compared to the other cell lines, C4-2B expressed intermediate miR-375 levels (Fig. 2B). The miR-375 level in C4-2B cells was increased by DHT stimulation in a dose-dependent manner (Fig. 2C), accompanied by reduced growth rate (*P* < 0.001) (Fig. 2D).

Thereby, the C4-2B cell line was chosen as a suitable model system based on its dominating MetB subtype at baseline and ability to regulate both miR-375 levels and similarity to MetA-C by androgen stimulation. Moreover, to mirror the MetB phenotype observed in clinical samples, an AR positive cell line was preferred over the AR negative cell lines.

### miRNA-375 suppresses MetB characteristics in C4-2B cells

**Fig. 3:**
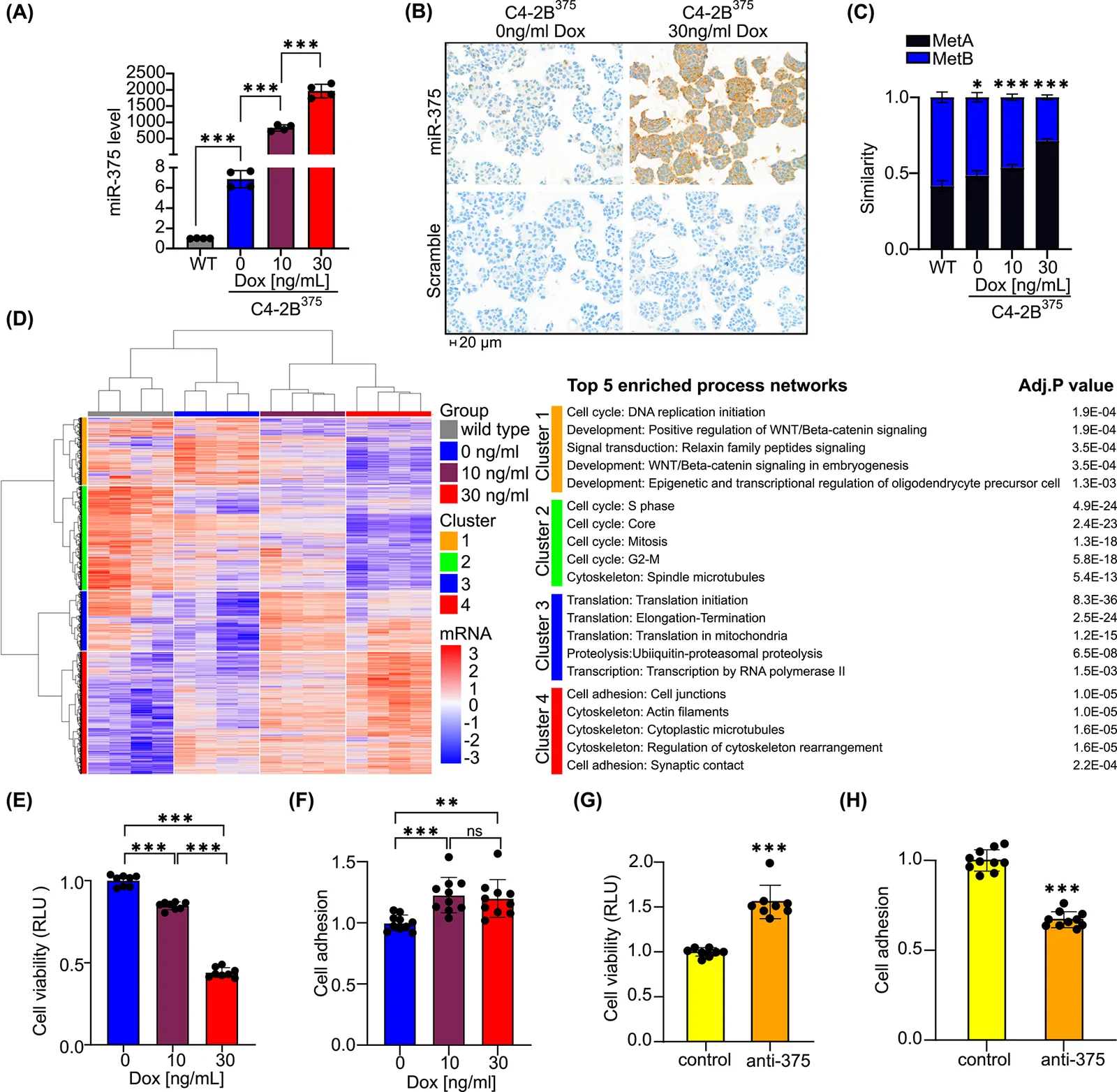
Overexpression of miR-375 shifts C4-2B cells towards less aggressive phenotype. (**A**) Relative miR-375 levels in wildtype (WT) C4-2B and in C4-2B^375^ cells after induction with 0, 10 or 30 ng/mL doxycycline (Dox) for 4 days (qRT-PCR). (**B**) Cytoplasmic staining of miR-375 in C4-2B^375^ cells after induction (30 ng/mL Dox, 4 days) according to in situ hybridization with no staining observed for scramble control. (**C**) Similarity to the MetA and MetB subtypes of WT C4-2B and C4-2B^375^ cells induced to overexpress miR-375 as in A (Clariom D array analysis, 4 replicates, MetA-B classification based on 157 differentiating transcripts). (**D**) Heatmap of differentially expressed transcripts (n = 7656, adj.*P* ≤ 0.05) in WT C4-2B or C4-2B^375^ cells, with top five enriched process networks based on gene clusters 1–4 listed (adj.*P* ≤ 0.05, MetaCore). (E–F) C4-2B^375^ cells induced with 0, 10 or 30 ng/mL doxycycline (Dox) to overexpress miR-375. **(E)** Cell viability in 3D cultures assessed after 7 days (3D CellTiter-Glo™). (**F**) Cell adhesion measured after 4 days (Vybrant assay). (**G**–**H**) C4-2B cells expressing a miR sponge construct to inhibit miR-375 (anti-375). (**G**) Cell viability in 3D cultures after 5 days (3D CellTiter-Glo™). (**H**) Cell adhesion after 4 days (Vybrant assay). Bars indicate mean ± SD, individual values shown. RLU, relative luminescence units. *P < 0.05, **P < 0.01, ***P < 0.001 (unpaired t-test). Data is shown from one representative experiment with replicates as indicated; experiments were independently repeated 3 (**E**–**F**) and 2 (**G**–**H**) times with similar results.

To functionally explore miR-375, C4-2B cells were transduced with an inducible miR-375 system, generating the C4-2B^375^ clone. Basal miR-375 levels were 6-fold higher in C4-2B^375^ compared to WT C4-2B, and Dox further induced miR-375 expression up to ~ 200-fold (*P* < 0.001) (Fig. 3A), which was confirmed by in situ hybridization showing strongly induced cytoplasmatic staining (Fig. 3B).

Transcriptomic profiling and MetA-C classification of C4-2B^375^ revealed that miR-375 induction shifted C4-2B^375^ cells from ~ 60% MetB to ~ 70% MetA (*P* < 0.001) (Fig. 3C) (Fig. S3). Overall, genome-wide transcriptomic analysis of WT C4-2B and C4-2B^375^ cells +/− Dox, identified 7656 differentially expressed transcripts (adj.*P* < 0.05 between at least two groups), including 1058 predicted and 120 stringently predicted miR-375 targets (Table S5). Unsupervised cluster analysis based on the 7656 differentially expressed genes revealed a clear separation of the sample groups by four gene clusters (Fig. 3D). Pathway analysis based on gene clusters 1–4 indicated reduced cycle activity (cluster 1–2), increased translation (cluster 3), and increased cell adhesion (cluster 4) upon miR-375 induction (Fig. 3D). Cluster 2 and 4 highlighted consistent miR-375-dependent changes from WT to C4-2B^375^, linking miR-375 to reduced proliferation and increased adhesion (Fig. 3D). Despite the overall MetB-to-MetA shift by miR-375 induction, no increase in AR signaling was observed (Fig. 3D).

Induction of miR-375 was confirmed to reduce cell growth (*P* < 0.001) (Fig. 3E) and enhance matrix adhesion in C4-2B^375^ cells (*P* < 0.01) (Fig. 3F), reflecting the pathway changes seen in clusters 2 and 4. Conversely, C4-2B cells expressing a miR-375 inhibitor sponge (anti-375) grew faster (Fig. 3G) and showed reduced adhesion (*P* < 0.001) (Fig. 3H).

**Fig. 4:**
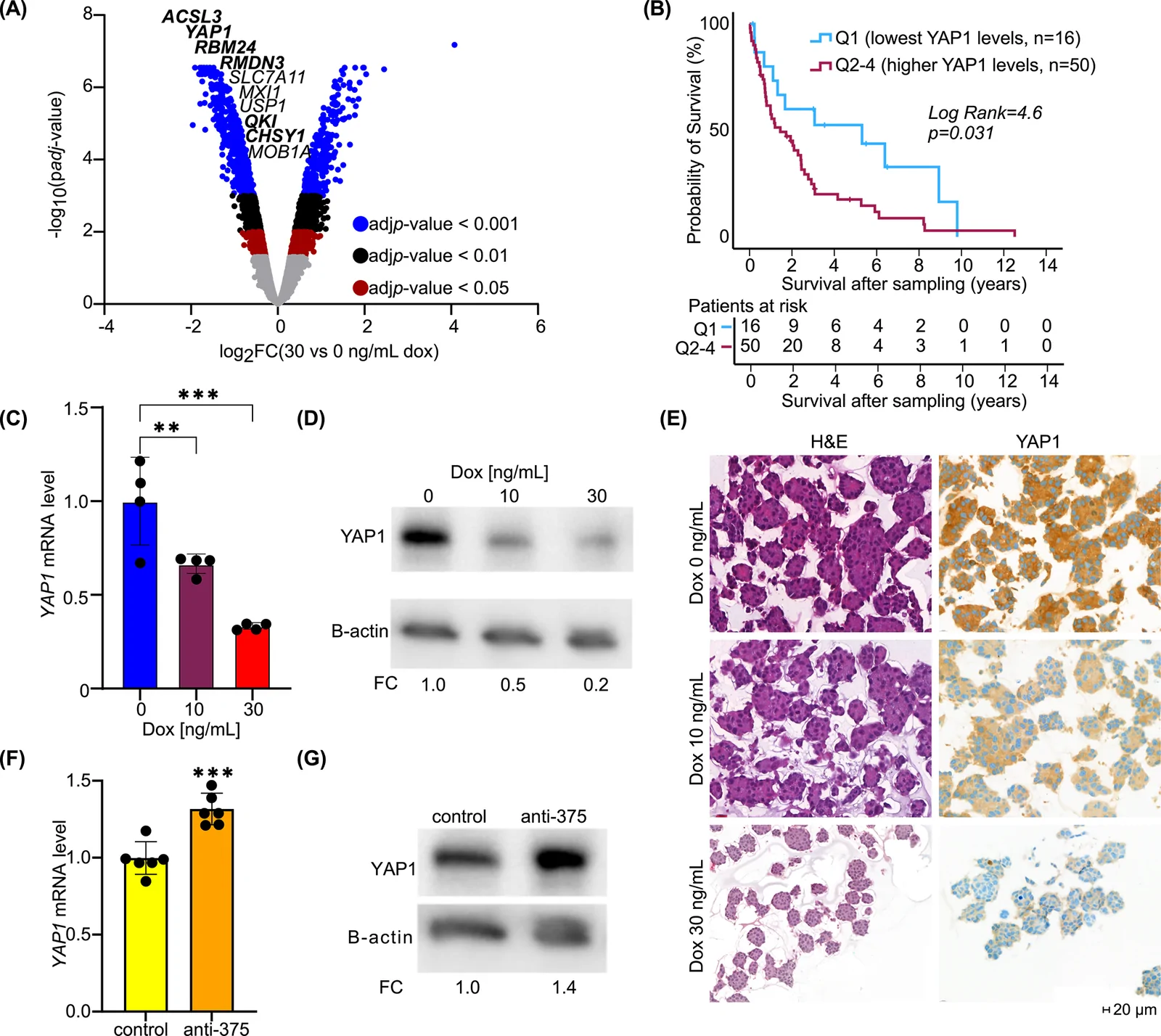
YAP1 as a marker for miR-375 effects in C4-2B cells, and its association with poor prognosis in metastatic patients. (**A**) Vulcano plot of differentially expressed genes in C4-2B^375^ after miR-375 induction (30 ng/ml Dox, 4 days) with top 10 stringently predicted miR-375 targets (Target scan) indicated (bold, top targets in common with 10 ng/ml Dox, Table S5). (**B**) Kaplan–Meier survival analysis of *YAP1* mRNA levels in metastasis samples, dichotomized into quartile 1 (Q1: the lowest 25^th^ percentile) and Q2-4, in relation to patient survival. (**C**–**E**) C4-2B^375^ cells induced with 0, 10 or 30 ng/mL doxycycline (Dox) for 7 days to overexpress miR-375. (**C**) *YAP1* mRNA levels (qRT-PCR). (**D**–**E**) YAP1 protein levels (Western blot and immunohistochemistry). (**F**–**G**) C4-2B cells expressing a miR sponge construct to inhibit miR-375 (anti-375). (**F**) *YAP1* mRNA (qRT-PCR) and (**G**) YAP 1 protein levels (Western blot) after 5 days in culture. Bars indicate mean ± SD, individual values shown. FC, fold-change (β-actin normalized). H&E, hematoxylin–eosin. **P < 0.01, ***P < 0.001 (unpaired t-test). Data is shown from one representative experiment with replicates as indicated; experiments were independently repeated 3 (**C**–**E**) and 2 (**F**–**G**) times with similar results. Original Western blots are presented in Fig. S6.

The *ACSL3, YAP1, RBM24, RMDN3, QKI, and CHSY1* gene transcripts stood out as being among the top downregulated, stringently predicted miR-375 targets after induction both with 10 and 30 ng/mL Dox (Fig. 4A) (Table S5). Of these genes, low levels of *YAP1* (Fig. 4B) or *RMDN3* (data not shown) were associated with better prognosis in the metastasis cohort. Based on this and the potential of *YAP1* as a regulator cell proliferation, migration, and invasion ^13,19^, *YAP1* was selected as a marker for miR-375 effects. Accordingly, *YAP1* was downmodulated at both mRNA (*P* < 0.01) (Fig. 4C) and protein levels (Fig. 4D–E) upon miR-375 induction, while miR-375 inhibition was accompanied by increased *YAP1* mRNA (*P* < 0.001) (Fig. 4F) and protein levels (Fig. 4G) compared to control. Control experiments confirmed that Dox did not affect cell growth, miR-375 or *YAP1* levels in WT C4-2B, and induction of NTC did not affect growth of C4-2B^375^ (Fig. S4).

### miR-375 inhibits tumor growth and suppresses YAP1 expression in vivo

To test the in vivo role of miR-375, C4-2B^375^ cells were subcutaneously injected into nude mice. Tumors developed in 12/31 mice (39%) after 4–8 weeks, compared to 6/7 mice (86%) within 3 weeks for WT C4-2B cells (*P* = 0.024), consistent with the ~ 6-fold higher basal level of miR-375 in C4-2B^375^.

**Fig. 5:**
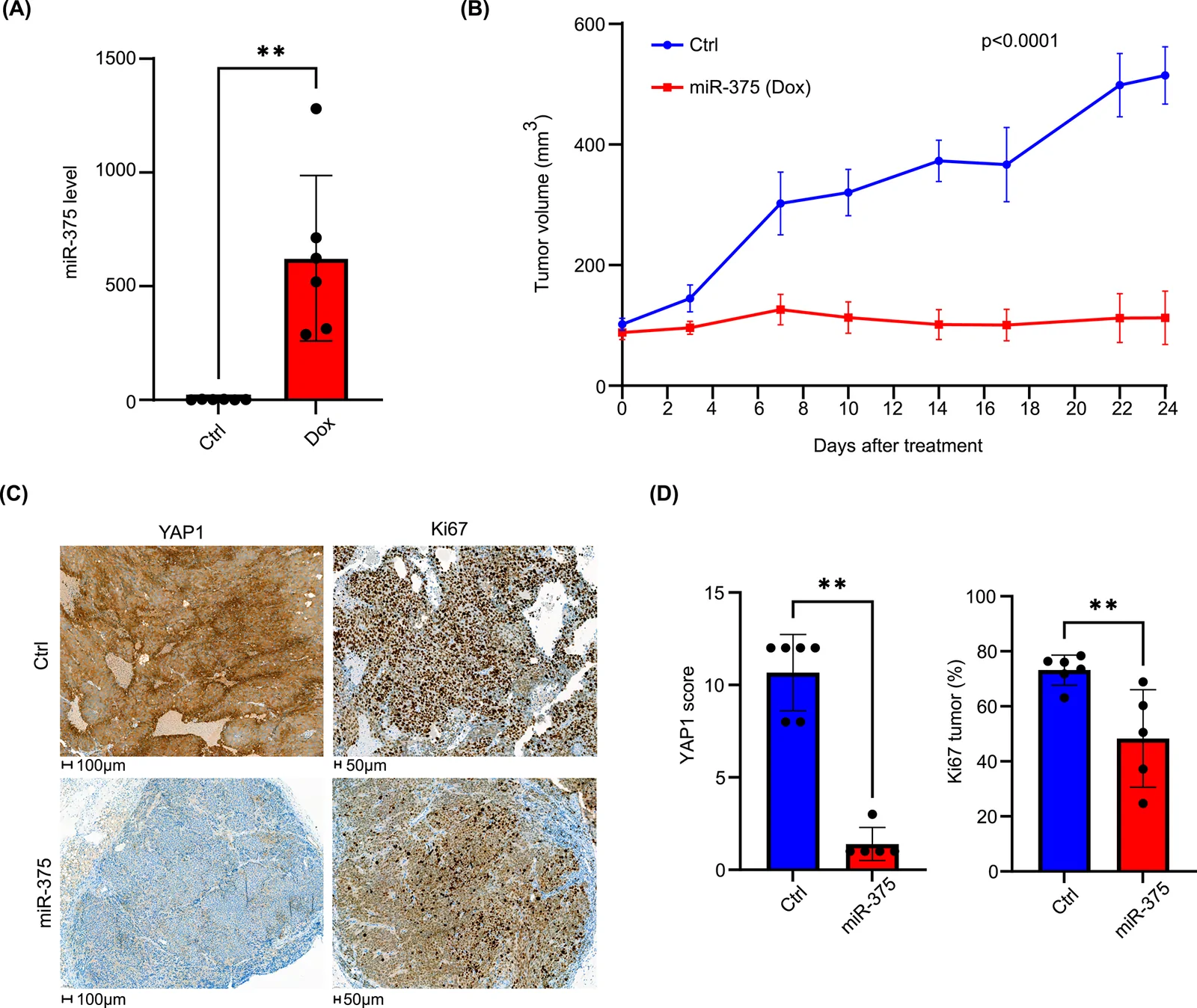
Overexpression of miR-375 inhibits tumor growth in mice. C4-2B^375^ tumors were grown subcutaneously in mice and miR-375 expression was induced with doxycycline (Dox) in drinking water (n = 6) or left non-induced in controls (n = 6). (**A**) miR-375 expression at the end of experiment in non-induced (Ctrl) and induced (Dox) tumors. (**B**) Tumor growth inhibition by miR-375 overexpression (Dox) in comparison to controls. (**C**) Representative images of immunoreactivity of YAP1 and Ki67 in C4-2B^375^ tumor with miR-375 overexpression and in control. (**D**) Quantification of YAP1 immunoreactivity and percentage of Ki67-positive tumor cells. Bars indicate mean ± SD, individual values shown. **P < 0.01 (unpaired t-test). Tumor growth over time is shown by mean ± SEM, P < 0.0001 (mixed model analysis).

When C4-2B^375^ tumors reached ~ 100 mm^3^, mice were randomized into non-induced control group (n = 6, control water) or miR-375-induced group (n = 6, Dox-water). Induction resulted in ~ 600-fold increased tumor levels of miR-375 compared to controls (*P* < 0.01) (Fig. 5A) and suppressed tumor growth (*P* < 0.0001) (Fig. 5B, Fig. S5). This was associated with reduced YAP1 immunostaining and fewer Ki67-positive tumor cells (*P* < 0.01) (Fig. 5C–D). Non-induced C4-2B^375^ tumors showed steady tumor growth and were euthanized within 24 days due to weight loss (Fig. S5).

Control experiments in WT C4-2B tumor-bearing mice confirmed that Dox did not affect tumor growth nor miR-375 or *YAP1* expression (Fig. S5), indicating a role for miR-375 in mediating the observed anti-tumor effects.

## Discussion

This study explores miR profiles of PC bone metastases in relation to the recently identified MetA-C subtypes^2,3^, and identifies miR-375 as an androgen-regulated miR downregulated in the highly proliferative and AR-deregulated MetB subtype. Overexpression of miR-375 reverted C4-2B cells from a MetB-like to a less aggressive MetA-like phenotype, primarily by reducing cell proliferation and increasing cell adhesion, whereas miR-375 inhibition produced opposite effects. In vivo, miR-375 suppressed tumor-take and growth, demonstrating strong tumor-inhibiting potential of miR-375 in aggressive PC.

miR-375 has been implicated as a tumor suppressor in multiple types of cancers^20^ and as regulator of epithelial–mesenchymal plasticity^13,14^. The growth inhibition and increased cell adhesion observed following miR-375 overexpression in the current study, together with the reverse effects observed after miR-375 inhibition, are consistent with a tumor-suppressive role for miR-375 in PC. Furthermore, these findings suggest that miR-375 may promote mesenchymal-to-epithelial transition, although confirmation of this hypothesis would require dedicated migration and invasion experiments. Although our results are in line with previous reports describing miR-375 as an inhibitor of cell proliferation, migration, and invasion in PC, both in C4-2B cells and in other aggressive PC cell lines^13,21,22^, conflicting results have also been reported, including increased miR-375 expression in clinically aggressive tumors and tumor-promoting effects of miR-375 in PC cell lines^21,23–26^. The reasons for this discrepancy are not clear, but it suggests that the functional role of miR-375 in PC may be context dependent.

Elevated expression and tumor-promoting effects miR-375 have primarily been associated with neuroendocrine PC (NEPC)^27,28^. However, we observed no association between miR-375 expression and the neuroendocrine markers *CHGA, SYP,* or *ENO2* in our metastasis cohort (data not shown). Furthermore, overexpression of miR-375 in C4-2B cells did not affect the expression of any of these neuroendocrine markers. We also found no relationship between the MetA–C subtypes and the NEPC phenotype, as determined by immunohistochemical analysis of CHGA^2,29^. Notably, the overall frequency of NEPC in our cohort was low^29^, likely reflecting the limited use of second-generation AR inhibitors in these cases. Therefore, we cannot exclude the possibility that a potential association among miR-375, the MetA–C subtypes, and NEPC was not detected because of the limited number of NEPC cases.

Many studies show higher miR-375 levels in PC versus benign prostate tissue^21,30,31^. This may reflect high tumor burden rather than high cellular levels, similar to what is seen for the well-known PC biomarker PSA that despite reduced levels with cellular dedifferentiation shows high levels in bulk tissue samples and in plasma. High miR-375 levels in liquid biopsies from PC patients^32–39^ may thus be merely related to high tumor volume (high T stage and M1) and not to high cellular levels.

Mechanistically, miR-375 directly targets expression of the transcriptional co-activator *YAP1*^13^, a regulator of proliferation, migration, and invasion^40^. Downregulation of *YAP1* is known to reduce growth of C4-2B xenografts in mice^41^, and *YAP1* downregulation upon miR-375 overexpression likely contributes to the tumor-inhibiting effects of miR-375 observed in the current study. Other miR-375 targets repressed here, including *ACSL3, RBM24, RMDN3, QKI,* and *CHSY1*, have been implicated in lipid metabolism, steroidogenesis, RNA regulation, and tumorigenesis^14,42–45^ and represent additional candidates for functional evaluation in metastatic PC. Off note, adverse prognosis was associated with high *RMDN3* mRNA levels (data not shown) and deserves further exploration for prognostic and therapeutic reasons. *RMDN3* has recently been described as a tethering factor between mitochondria and endoplasmic reticulum, facilitating cell survival through the removal of lipid radicals, but little is known about its relevance for PC^45,46^. Besides downregulation of miR-375 in MetB, we found low levels of miR-130b-3p, -1180-3p, -1290, -1269b, -433-3p, and -210-3p in MetA. Those miRs have been previously associated with PC^47–51^, but their potential roles as regulators of MetA–MetB plasticity warrant further exploration.

The C4-2B cell line was selected as a model system to study the effects of miR-375 on MetB-to-MetA plasticity based on its predominant MetB phenotype at baseline and its ability to regulate both miR-375 expression and subtype in response to DHT stimulation. Furthermore, an AR-positive model system was preferred over AR-negative models, as the MetB subtype generally exhibits low AR activity despite retained AR expression^2^. In WT C4-2B cells, miR-375 expression was induced by DHT stimulation, suggesting that miR-375 is androgen regulated. It is therefore plausible that miR-375 contributes to the reduced cell proliferation observed in C4-2B cells following DHT treatment. Androgen-mediated inhibition of cell proliferation has also been demonstrated in the LNCaP cell line^52^ and may reflect mechanisms involved in normal prostate homeostasis, where AR signaling suppresses proliferation and promotes terminal differentiation of luminal epithelial cells^53^.

## Conclusions

The miR-375 is heterogeneously expressed in PC bone metastasis and is inversely associated with the recently identified, highly proliferative MetB subtype. Forced overexpression of miR-375 in C4-2B tumor cells inhibits aggressive MetB-like traits and promotes less aggressive MetA-like tumor characteristics, including reduced cell proliferation, increased cell adhesion, and suppressed tumor growth in mice. Collectively, these findings link clinical and experimental evidence supporting miR-375 as a potential regulator of metastasis subtype in PC and a potential therapeutic candidate for metastatic PC of the MetB subtype. This study is limited by its reliance on a single experimental model system and the need for validation in independent patient cohorts.

## Supplementary Information

Below is the link to the electronic supplementary material.

Supplementary Material 1

Supplementary Material 2

Supplementary Material 3

Supplementary Material 4

Supplementary Material 5

Supplementary Material 6

## Data Availability

The data sets generated from cell lines during the study are freely available in NCBI’s Gene Expression Omnibus at [https://www.ncbi.nlm.nih.gov/geo/], reference numbers [GSE272445], [GSE272448], and [GSE272451]. The study also made use of the previously generated data set [GSE189343]. Other data generated and analyzed in the current study is not publicly available due to Swedish laws and regulations but is available from the corresponding author upon reasonable request.

## Declarations

### AI declaration

During the preparation of this work the authors used ChatGPT (openai.com) to improve language and readability. After using this tool, the authors reviewed and edited the content as needed and take full responsibility for the content of the publication.
