# Supplementary material for: MicroRNA profiles of prostate cancer bone metastases and miR-375 as an inhibitor of the aggressive metastasis subtype MetB
Source: Sci Rep. 2026 Aug 25;16:26685. doi: 10.1038/s41598-026-67032-9 (PMC13507049; doi:10.1038/s41598-026-67032-9)
Supplement: Supplementary file 1 [file 41598_2026_67032_MOESM1_ESM.pdf]

SUPPLEMENTARY FIGURES

Järemo et al.

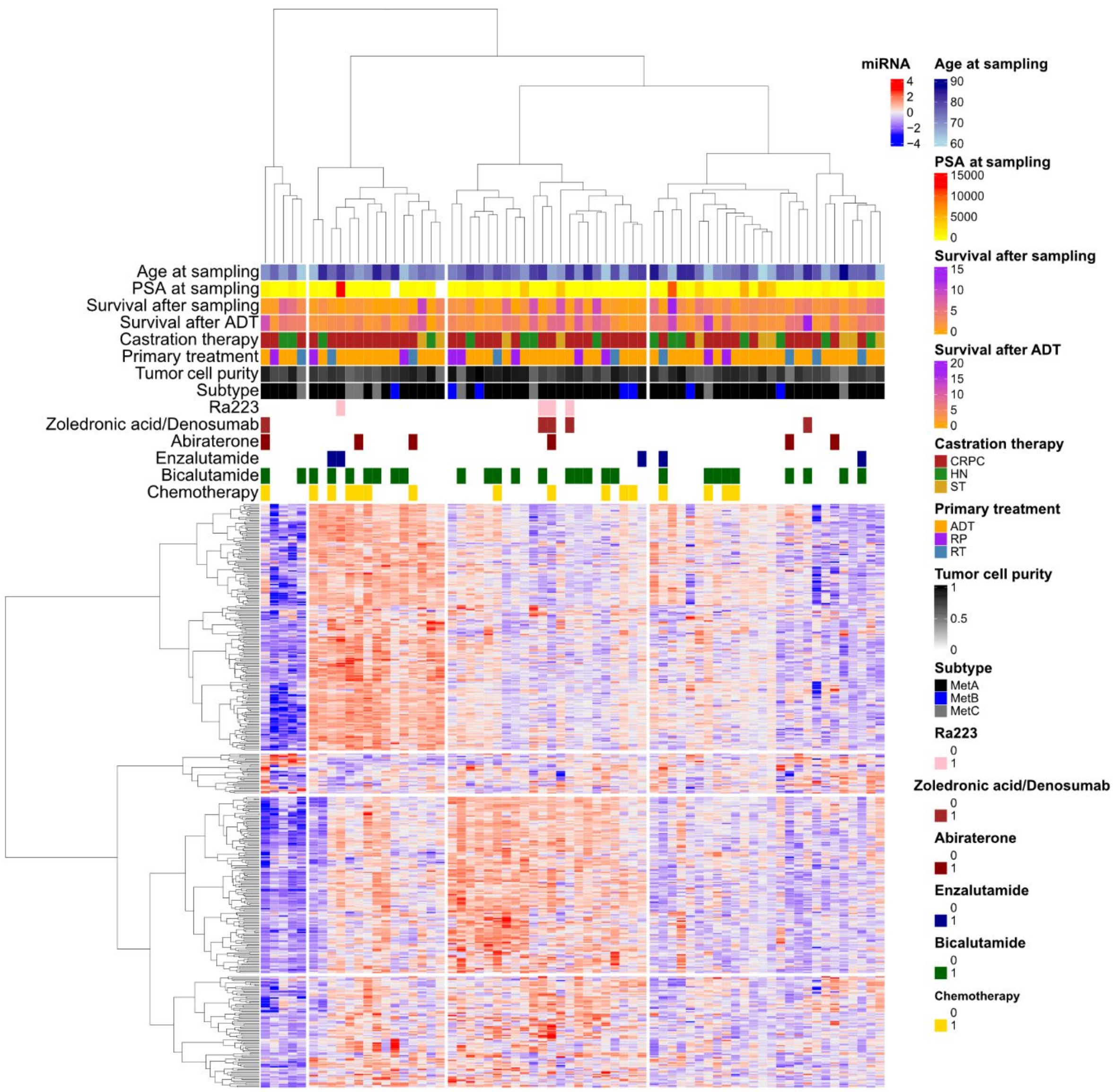

Figure S1. Heatmap displaying unsupervised clustering of 62 patients based on metastasis levels of 459 miRs. Clustering based on 459 significantly detected, highly variable miRs (GeneChip™ miRNA 4.0) revealed four patient clusters without clear association to the clinical variables examined (Table 1). Clustering was based on mean miR levels per patient. Abbreviations: CRPC, castration-resistant prostate cancer; HN, hormone naïve; ST, short-term treatment; ADT, androgen-deprivation therapy; RP, radical prostatectomy; RT, radiotherapy.

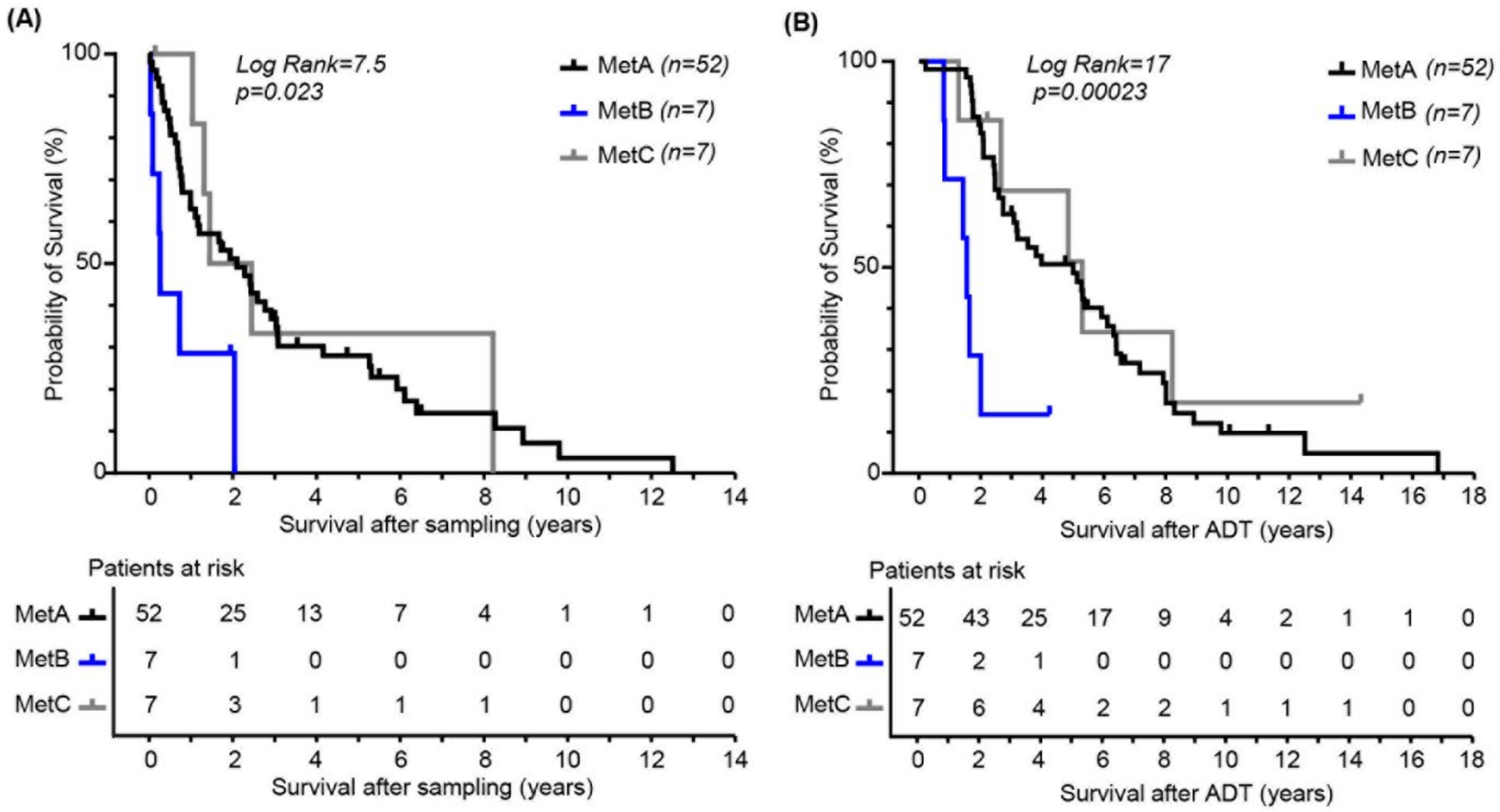

Figure S2. Metastasis subtypes MetA-C and cancer-specific survival in 62 patients with bone metastatic prostate cancer. Kaplan-Meier survival analysis showing the association between metastasis subtypes (MetA-C) and cancer-specific survival after (**A**) metastasis tissue sampling, and (**B**) initiation of androgen-deprivation therapy (ADT).

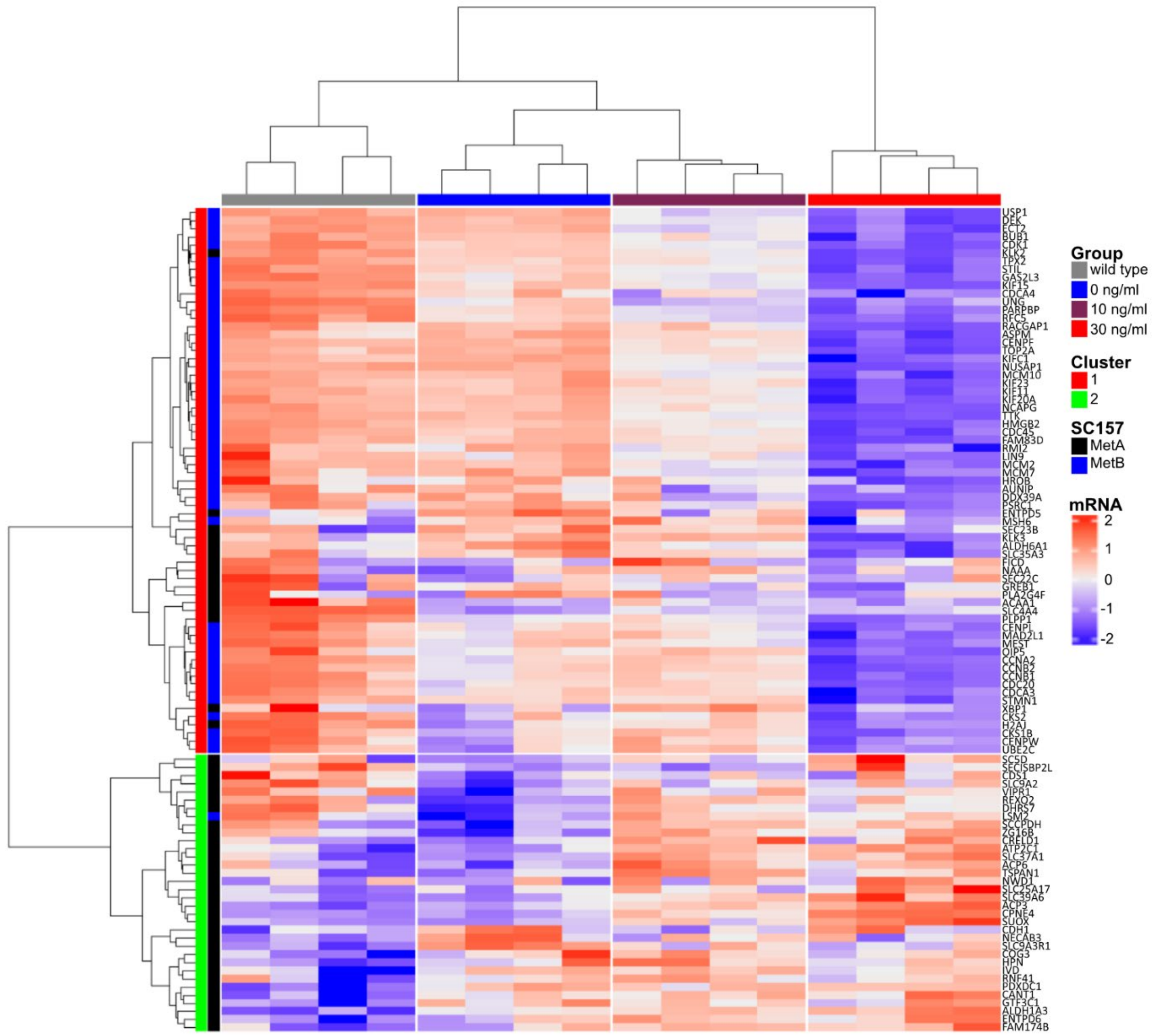

Figure S3: Heatmap displaying unsupervised clustering of wildtype (WT) C4-2B and C4-2B^{375} cells after miR-375 induction with doxycycline. Clustering of C4-2B^{375} cells following induction of miR-375 by doxycycline (10 and 30 ng/mL) for 4 days as compared to non-induced (0 ng/ml) and wild-type C4-2B cells, based on transcript levels (Clariom D arrays) of predefined MetA- and MetB-differentiating genes (as indicated).

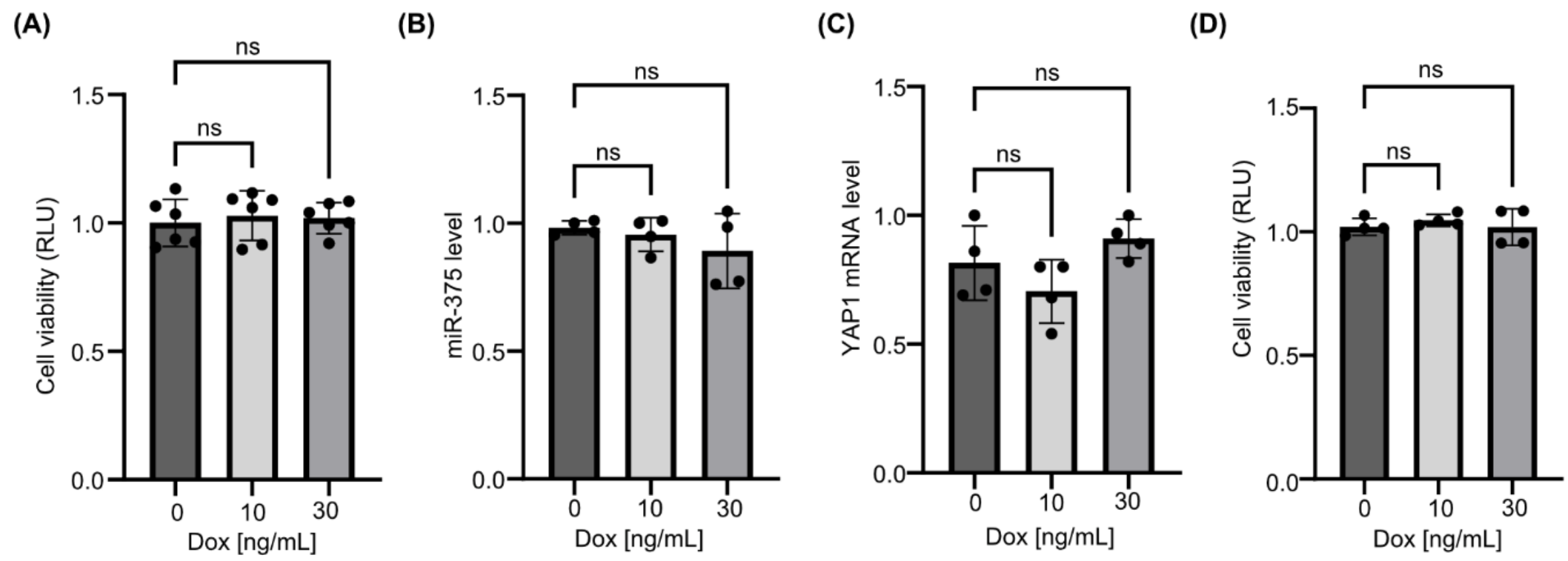

Figure S4: No effect of doxycycline on wild-type (WT) C4-2B cells or C4-2B cells transduced with a non-target control (NTC) *in vitro*. (A-C) WT C4-2B cells cultured and treated with 0, 10 or 30 ng/mL doxycycline (Dox) for four days. (**A**) Relative cell growth in 2D cultures (2D CellTiter-Glo). (**B-C**) Relative expression of miR-375 and YAP1 (qRT-PCR). (**D**) Relative cell growth of C42B cells transduced with NTC and treated with Dox. Bars indicate mean ± SD, individual values shown. ns, $P < 0.05$ (unpaired t-test).

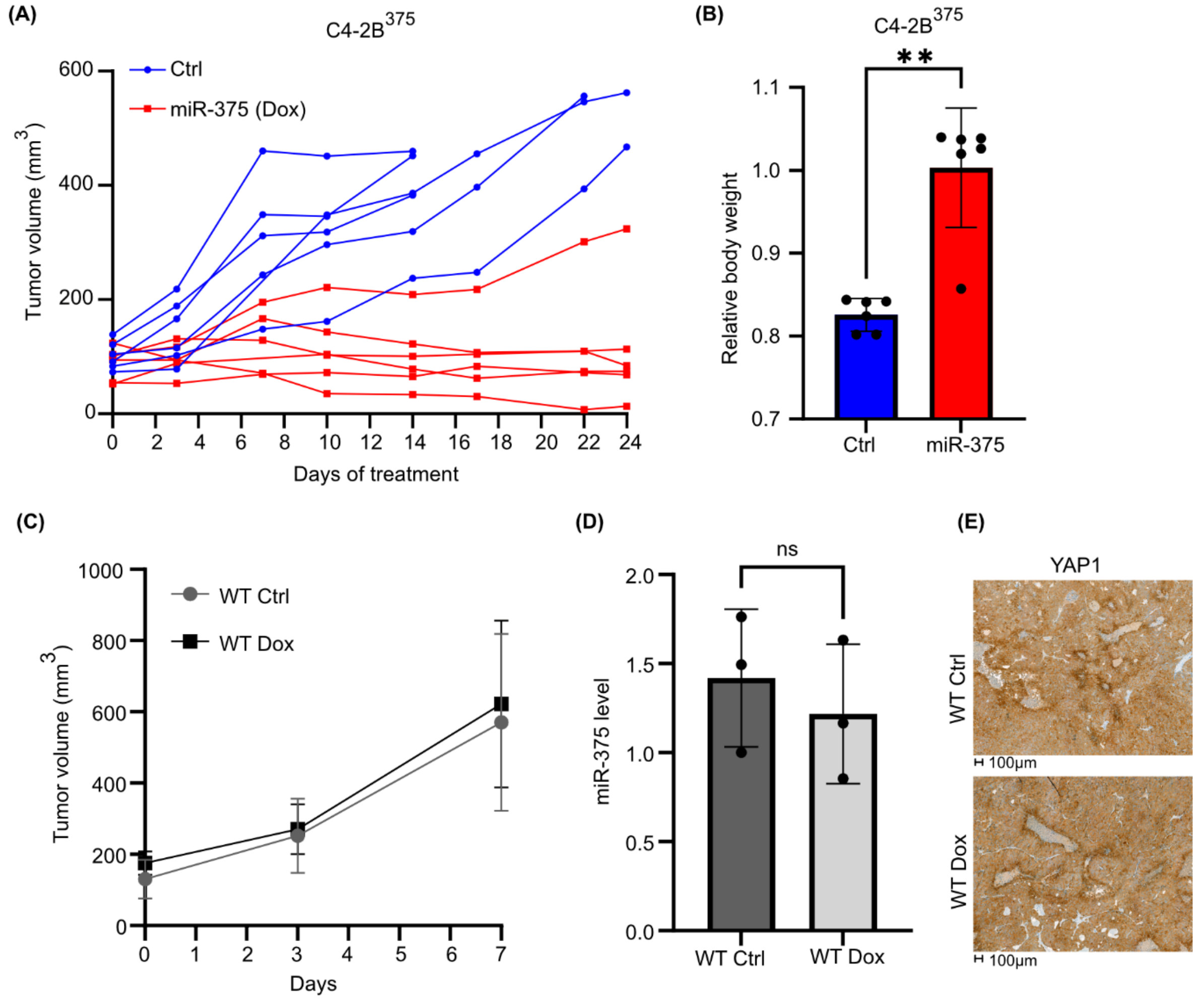

Figure S5. miR-375 overexpression inhibits tumor growth in mice. (A) Doxycyclin (Dox) given in the drinking-water to mice with subcutaneous C4-2B^{375} tumors (n=6) inhibits tumor growth in comparison to non-induced C4-2B^{375} controls (n=6). Individual tumor volumes are shown. (**B**) Control mice with C4-2B^{375} tumors without receiving Dox showed reduced body weights at endpoint. (**C-E**) Dox given in drinking-water to mice with wild type (WT) C4-2B tumors (n=3) does not affect tumor growth (**C**) nor expression levels of miR-375 (**D**), or *YAP1* (**E**) in comparison to control WT mice not receiving Dox (n=3). Bars indicate mean ± SD, individual values shown. $^{**}P<0.01$, ns, $P > 0.05$ (unpaired t-test).

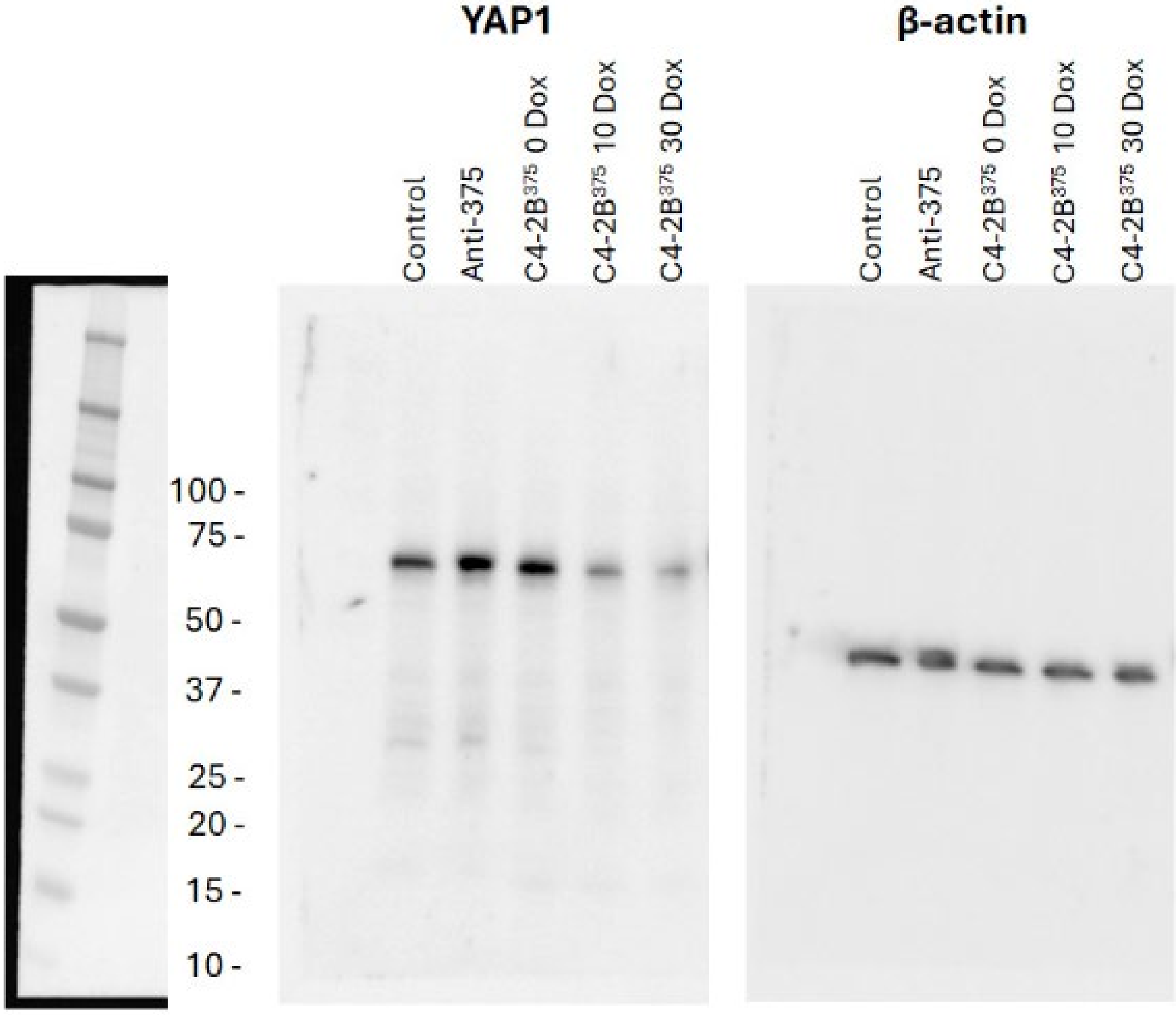

Figure S6. Original Western blots corresponding to the cropped blots shown in Figure 4D and G. YAP1 and β-actin protein levels assessed by Western blot analysis of C4-2B^{375} cells induced with 0, 10 or 30 ng/mL doxycycline (Dox) to overexpress miR-375 and of C4-2B cells transduced with a miR sponge construct to inhibit miR-375 (anti-375). The molecular weights of YAP1 (65 kDa) and β-actin (42 kDa) were assured by parallelled analysis of Precision Plus Protein Dual Color Standards (BIO-RAD).
